# Updated results from the phase 3 HELIOS study of ibrutinib, bendamustine, and rituximab in relapsed chronic lymphocytic leukemia/small lymphocytic lymphoma

**DOI:** 10.1038/s41375-018-0276-9

**Published:** 2018-10-12

**Authors:** G. Fraser, P. Cramer, F. Demirkan, R. Santucci Silva, S. Grosicki, A. Pristupa, A. Janssens, J. Mayer, N. L. Bartlett, M.-S. Dilhuydy, H. Pylypenko, J. Loscertales, A. Avigdor, S. Rule, D. Villa, O. Samoilova, P. Panagiotidis, A. Goy, M. A. Pavlovsky, C. Karlsson, M. Hallek, M. Mahler, M. Salman, S. Sun, C. Phelps, S. Balasubramanian, A. Howes, A. Chanan-Khan

**Affiliations:** 1 Juravinski Cancer Centre, McMaster University, Hamilton, ON Canada; 2 Department of Internal Medicine, Center of Integrated Oncology and German CLL Study Group, University of Cologne, Cologne, Germany; 3 Division of Hematology, Dokuz Eylul University, Izmir, Turkey; 4 IEP São Lucas/Hemomed Oncologia e Hematologia, São Paulo, Brazil; 5 Department of Cancer Prevention, Faculty of Public Health, Silesian Medical University, Katowice, Poland; 6 Regional Clinical Hospital, Ryazan, Russia; 7 Universitaire Ziekenhuizen Leuven, Leuven, Belgium; 8 Department of Internal Medicine, Hematology and Oncology, Masaryk University Hospital Brno, Jihlavska, Brno, Czech Republic; 9 Siteman Cancer Center, Washington University School of Medicine, St Louis, MO USA; 10 Hôpital Haut-Lévêque, Pessac, Bordeaux, France; 11 Department of Hematology, Cherkassy Regional Oncological Center, Cherkassy, Ukraine; 12 Hematology Department, Hospital Universitario La Princesa, IIS-IP, Madrid, Spain; 13 Division of Hematology and Bone Marrow Transplantation, Chaim Sheba Medical Center, Tel-Hashomer and Sackler School of Medicine, University of Tel-Aviv, Tel-Aviv, Israel; 14 Department of Haematology, Plymouth University Medical School, Plymouth, UK; 15 Division of Medical Oncology, British Columbia Cancer Agency, Vancouver, BC Canada; 16 Nizhny Novogorod Regional Clinical Hospital, Nizhny Novogorod, Russia; 17 1st Department of Propedeutic Medicine, National and Kapodistrian University of Athens, Athens, Greece; 18 John Theurer Cancer Center at Hackensack University Medical Center, Hackensack, NJ USA; 19 Department of Hematology, Fundaleu, Buenos Aires, Argentina; 20 Department of Hematology, Karolinska University Hospital, Stockholm, Sweden; 21 Department of Oncology-Pathology, Karolinska Institutet, Stockholm, Sweden; 22 Department I of Internal Medicine, University of Cologne, Cologne, Germany; 23 Janssen Research & Development, Raritan, NJ USA; 24 Janssen Research & Development, Spring House, PA USA; 25 Janssen Research & Development, High Wycombe, UK; 26 Mayo Clinic Cancer Center, Jacksonville, FL USA

**Keywords:** Chronic lymphocytic leukaemia, Chronic lymphocytic leukaemia, Molecularly targeted therapy

## Abstract

We report follow-up results from the randomized, placebo-controlled, phase 3 HELIOS trial of ibrutinib+bendamustine and rituximab (BR) for previously treated chronic lymphocytic leukemia (CLL)/small lymphocytic lymphoma (SLL) without deletion 17p. Overall, 578 patients were randomized 1:1 to either ibrutinib (420 mg daily) or placebo, in combination with 6 cycles of BR, followed by ibrutinib or placebo alone. Median follow-up was 34.8 months (range: 0.1–45.8). Investigator-assessed median progression-free survival (PFS) was not reached for ibrutinib+BR, versus 14.3 months for placebo+BR (hazard ratio [HR] [95% CI], 0.206 [0.159–0.265]; *P* < 0.0001); 36-month PFS rates were 68.0% versus 13.9%, respectively. The results are consistent with the primary analysis findings (HR = 0.203, as assessed by independent review committee, with 17-month median follow-up). Median overall survival was not reached in either arm; HR (95% CI) for ibrutinib+BR versus placebo: 0.652 (0.454–0.935; *P* = 0.019). Minimal residual disease (MRD)-negative response rates were 26.3% for ibrutinib+BR and 6.2% for placebo+BR (*P* < 0.0001). Incidence of treatment-emergent adverse events (including grades 3–4) were generally consistent with the initial HELIOS report. These long-term data support improved survival outcomes and deepening responses with ibrutinib+BR compared with BR in relapsed CLL/SLL.

## Introduction

Ibrutinib is an oral, once-daily inhibitor of Bruton’s tyrosine kinase, an essential enzyme in the B cell receptor signaling pathway [1–3]. The efficacy and safety of ibrutinib has been demonstrated in patients with chronic lymphocytic leukemia (CLL) and small lymphocytic lymphoma (SLL) in treatment-naive and relapsed/refractory settings [4, 5], leading to approvals for these indications [6, 7]. Ibrutinib as a single agent for previously treated patients with CLL/SLL was evaluated in a phase 1b/2 study (Study 1102 and its extension, Study 1103) and the phase 3 RESONATE study of ibrutinib versus ofatumumab [8, 9]. Long-term follow-up data from these studies showed that continuing ibrutinib treatment leads to durable and deepening responses. The phase 1b/2 study (101 patients with previously treated CLL) reported an overall response rate (ORR) of 89% with 10% complete responses (CRs) and a median progression-free survival (PFS) of 52 months after 5-year follow-up, while the median overall survival (OS) remained unreached [9]. In the RESONATE™ study (195 previously treated CLL patients), the ORR was 91% (with 9% CR/CRi [CR with incomplete bone marrow recovery]) at a median follow-up of 44 months versus 83% (2% CR/CRi) after median follow-up of 9.4 months [8].

Chemoimmunotherapy regimens such as bendamustine and rituximab (BR) or fludarabine, cyclophosphamide, and rituximab (FCR) are efficacious in patients with relapsed/refractory CLL, but their use is often limited by patient tolerability [10]. BR has been commonly used [11], largely based on a phase 2 study in relapsed/refractory CLL that showed an ORR of 59%, with 9% of patients achieving a CR, and a median PFS and OS of 15 and 34 months, respectively [12]. The BR regimen formed the backbone of the phase 1b study that led to the development of the HELIOS study [13]. In this phase 1b study (Study 1108) with 30 previously treated patients receiving up to six cycles of BR+continuous ibrutinib, the CR rate was 17% after a median of 15.8 months of follow-up, increasing to 40% at a median follow-up of 37.3 months [13].

In the phase 3 HELIOS trial of 578 patients with relapsed/refractory CLL, ibrutinib+BR (≤6 cycles) significantly improved PFS at the initial analysis (median follow-up 17 months); median PFS was not reached in the ibrutinib arm versus 13.3 months in the placebo arm (hazard ratio [HR] = 0.203, 95% confidence interval (CI): 0.150–0.276; *P* < 0.0001) [14]. The findings of HELIOS supported the approval of ibrutinib+BR in the US and EU for patients with relapsed/refractory CLL/SLL [6, 7].

For traditional chemoimmunotherapy, minimal residual disease (MRD)-negative responses are prognostic for prolonged PFS [15] and may be a more potent predictor of PFS than the clinical response assessment according to International Workshop on Chronic Lymphocytic Leukemia (iwCLL) guidelines [16]. Because of limited long-term follow-up data on novel targeted therapies, it is unclear whether MRD-negative remissions are similarly prognostic in patients receiving these agents [17]. Evaluation of MRD status is of particular interest in ibrutinib-containing regimens, as MRD negativity represents a lower disease burden and is being investigated as a marker for treatment discontinuation with novel agents, which are usually administered until progression or unacceptable toxicity. HELIOS was the first study to evaluate MRD status in ibrutinib-treated patients. At 17-month median follow-up, the proportion of patients that achieved MRD negativity was higher with ibrutinib+BR versus placebo+BR (13% versus 5%; *P* = 0.0011) [14].

As ibrutinib is a continuously administered oral once-daily therapy, data addressing the safety profile of ibrutinib over time, longer-term outcomes, and efficacy in patient subgroups become increasingly relevant. We report updated data from HELIOS (3-year follow-up) to determine survival outcomes, evolution of responses, and durability of remissions across patient subgroups, as well as long-term safety.

## Subjects and methods

### Study design and patients

Study design and participants have been previously described [14]. Briefly, HELIOS (Clinicaltrials.gov #NCT01611090) is a phase 3, randomized, placebo-controlled, double-blind study of 578 patients conducted at 133 sites in 21 countries between September 19, 2012 and January 21, 2014. Eligible patients were aged ≥18 years, had a diagnosis of CLL/SLL according iwCLL criteria [18], relapsed/refractory disease following ≥1 previous lines of systemic therapy, an Eastern Cooperative Oncology Group performance status of 0–1, measurable lymph node disease (>1.5 cm) by computed tomography (CT) scan, and adequate liver and kidney function. Patients with deletion 17p (≥20% of blood or bone marrow cells examined by fluorescence in situ hybridization) were excluded owing to known poor response to BR.

Patients were randomly assigned 1:1 to ibrutinib (420 mg daily)+BR or placebo+BR. BR was administered for up to six cycles (bendamustine: 70 mg/m^2^ intravenously on days 2–3 in cycle 1 and days 1–2 in cycles 2–6; rituximab: 375 mg/m^2^ on day 1 of cycle 1 and 500 mg/m^2^ on day 1 of cycles 2–6). After 6 months of BR with ibrutinib or placebo therapy, patients continued ibrutinib treatment or placebo alone until disease progression or unacceptable toxicity. Following the pre-specified interim analysis, the study was unblinded and placebo treatment was discontinued. Subsequently, adverse events (AEs) were collected only for patients continuing on ibrutinib, although patients originally treated with placebo were followed with regular disease evaluations and were able to crossover to ibrutinib at the time of progression and meeting iwCLL criteria for treatment.

### End points and assessments

The primary end point was Independent Review Committee (IRC)-assessed PFS, for which results were reported previously [14]. Investigator-assessed end points were used for the follow-up analyses reported here. Key secondary end points were investigator-assessed PFS, OS, and response rates; proportion of patients with MRD-negative responses (<1 CLL cell per 10,000 leukocytes or <0.01%) confirmed by central laboratory assessment of peripheral blood or bone marrow aspirate; and safety. PFS2 (time interval from randomization to disease progression on next-line treatment or death or start of next antineoplastic therapy if no progressive disease [PD] was recorded) was also assessed.

Assessment of tumor response was conducted in accordance with iwCLL 2008 criteria [18]. Prior to the interim analysis, CT scans were performed at baseline, then every 12 weeks for 2 years and every 6 months thereafter. Following the interim analysis, disease evaluations based on the discretion of investigators continued every 3 months in both arms; for patients randomized into the ibrutinib arm who had not yet progressed, CT scans continued every 6 months until progression. Analysis of MRD was initially performed on bone marrow sampled at the time of radiological documentation of CR, with subsequent analyses of peripheral blood every 12 weeks. After the interim analysis, the protocol was amended to include MRD analysis for all patients with a partial response (PR) or better. Testing was performed at a central laboratory by flow cytometry using an eight-color panel of antibodies in keeping with the EuroFlow panel [19].

### Statistical analysis

Statistical analyses have been described previously [14]. Approximately 580 patients were randomized to observe 342 PFS events to detect an HR of 0.7 for the ibrutinib+BR group relative to the placebo+BR group with 90% power at a one-sided significance level of 0.025, using a group sequential testing design. The distribution of time-to-event end points was estimated using the Kaplan–Meier method.

The analysis of PFS and OS using the long-term follow-up data was similar to those used for the primary analyses, except that investigator assessments were used for follow-up data. For patients in the placebo+BR group who crossed over to receive ibrutinib, no adjustment was made for OS analysis, i.e., the OS is defined as the time interval from randomization to death irrespective of cause. For surviving patients, the OS is censored at the last date known to be alive. Separate analyses of OS corrected for crossover were performed using the inverse probability of censoring weighting and the rank preserving structural failure time methods (Supplementary Figure S1). The MRD-negative response rate was compared between treatment arms using Fisher’s exact test; MRD assessments continued until crossover for the placebo+BR arm.

## Results

### Study population

The data represent outcomes of 6 months of combination therapy (ibrutinib+BR or placebo+BR) followed by >2 years of continuous ibrutinib or placebo treatment. For consistency with the initial analysis, the treatment arms are referred to as ibrutinib+BR and placebo+BR. The median follow-up period at this analysis was 34.8 months (range: 0.1–45.8), with a median treatment duration of 34.7 months (range: 0.2–43.3) for ibrutinib+BR and 14.3 months (range: 0.2–30.6) months for placebo+BR (Supplementary Table S1). Sixty-six percent (188/287) of ibrutinib-treated patients remained on treatment for ≥24 months.

Patient disposition is shown in Table 1. A total of 160 (55.4%) patients who had confirmed PD in the placebo+BR arm crossed over to ibrutinib. At the time of this analysis, patients received crossover therapy for a median of 16.9 months (range: 0.2–26.3). Patient demographics and baseline characteristics data were previously reported and were balanced between arms (Supplementary table S2) [14].

**Table 1:** Patient disposition

Patient status, *n* (%)	Ibrutinib+BR (*n* = 289)	Placebo+BR (*n* = 289)	Total (*N* = 578)
Median months on study (95% CI)	35.1 (33.7–35.9)	34.5 (33.8–35.5)	34.8 (34.1–35.5)
Study treatment phase disposition, *n* (%)			
Did not receive study drug	2 (0.7)	2 (0.7)	4 (0.7)
Ongoing	171 (59.2)	0	171 (29.6)
Discontinued study treatment	116 (40.1)	287 (99.3)	403 (69.7)
Primary reason for discontinuation^a^			
Adverse event	47 (16.3)	34 (11.8)	81 (14.0)
Death	14 (4.8)	9 (3.1)	23 (4.0)
Lost to follow-up	1 (0.3)	1 (0.3)	2 (0.3)
Progressive disease or relapse	27 (9.3)	148 (51.2)	175 (30.3)
Investigator or sponsor decision	9 (3.1)	83 (28.7)	92 (15.9)
Withdrawal of consent	20 (6.9)	14 (4.8)	34 (5.9)
Follow-up phase disposition, *n* (%)			
In follow-up phase	46 (15.9)	194 (67.1)	240 (41.5)
Post-treatment, prior to follow-up visit^b,c^	4 (1.4)	1 (0.3)	5 (0.9)
Pre-progressive disease follow-up	14 (4.8)	47 (16.3)	61 (10.6)
Post-progressive disease follow-up	28 (9.7)	146 (50.5)	174 (30.1)
Crossover to ibrutinib^c^		160 (55.4)	
Death during crossover period		23 (8.0)	
Discontinued study	72 (24.9)	95 (32.9)	167 (28.9)
Primary reason for discontinuation			
Withdrawal of consent	16 (5.5)	21 (7.3)	37 (6.4)
Lost to follow-up	5 (1.7)	2 (0.7)	7 (1.2)
Death	51 (17.6)	72 (24.9)	123 (21.3)

*BR* bendamustine and rituximab, *CI* confidence interval

^a^Includes patients who did not receive study medication

^b^A patient is counted here if the patient discontinued treatment but did not discontinue the study and did not yet have a first follow-up visit at the time of clinical cutoff

^c^Crossover patients may also be counted under the “post-treatment, prior to follow-up visit” category

### Efficacy

**Fig. 1:**
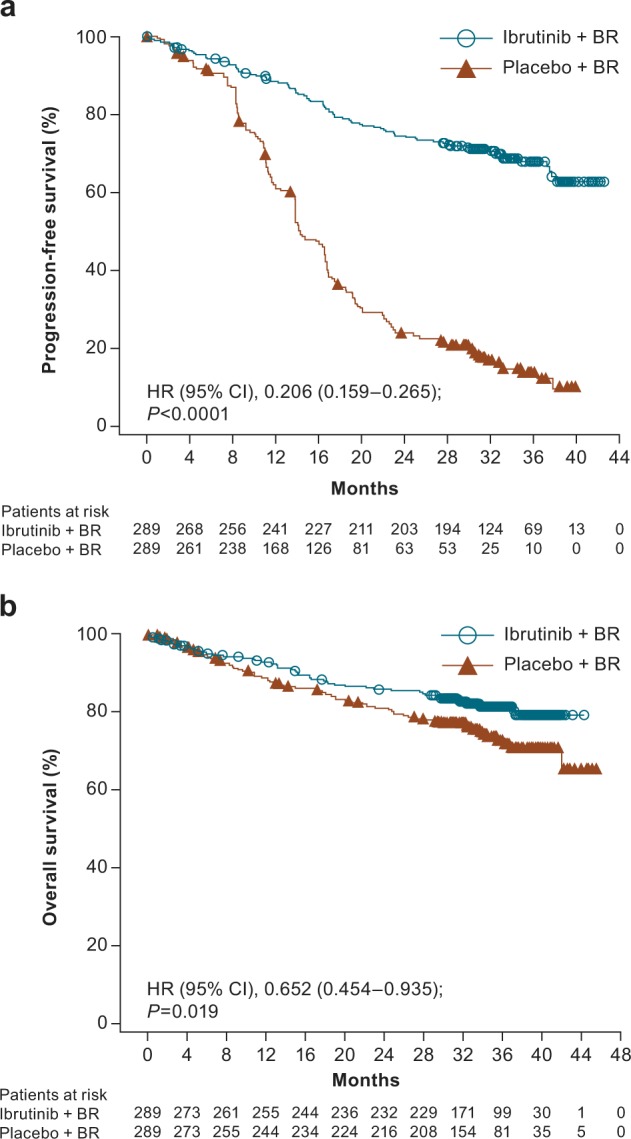
Three-year follow-up of investigator-assessed **a** progression-free survival and **b** overall survival. BR bendamustine and rituximab, CI confidence interval, HR hazard ratio, OS overall survival, PFS progression-free survival

Investigator-assessed PFS was significantly longer with ibrutinib+BR (not reached versus 14.3 months for placebo+BR [HR (95% CI), 0.206 (0.159–0.265); *P* < 0.0001]) (Fig. 1a), and the 36-month PFS rate was 68.0% versus 13.9%, respectively. Median OS was not reached in either arm but was significantly longer for the ibrutinib+BR arm (HR [95% CI], 0.652 [0.454–0.935]; *P* = 0.019) (Fig. 1b); the 36-month OS rate for each arm was 81.6% versus 72.9%, respectively. An analysis of OS that corrected for crossover from the placebo+BR arm to ibrutinib+BR confirmed the OS advantage of ibrutinib+BR (Supplementary Figure S1).

**Fig. 2:**
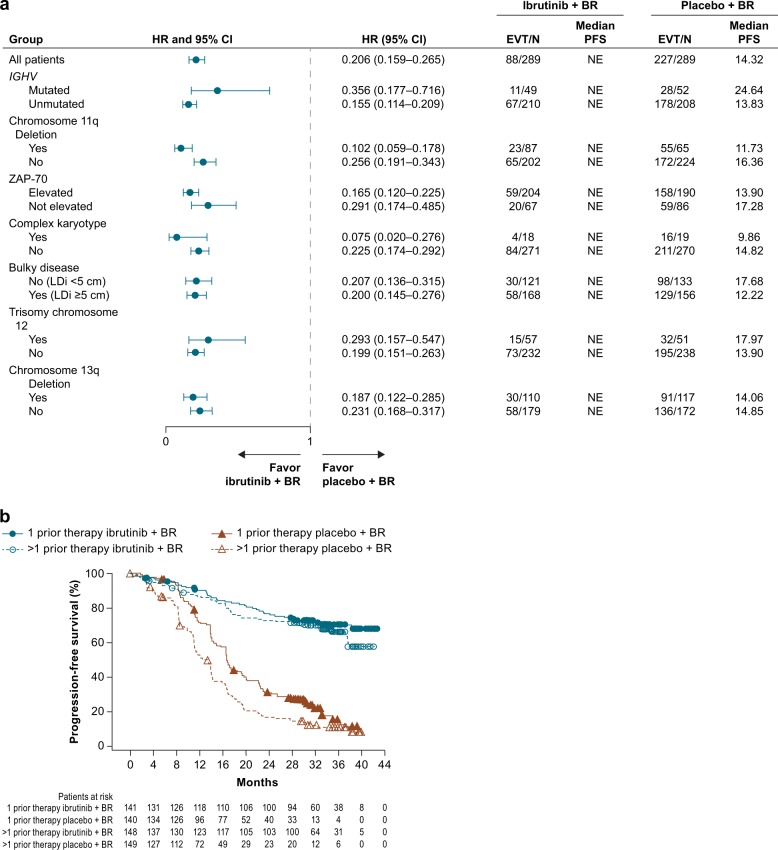
Investigator-assessed PFS by **a** prognostic factors and **b** prior lines of therapy. BR bendamustine and rituximab, CI confidence interval, EVT event, *IGHV* immunoglobulin heavy-chain variable, HR hazard ratio, LDi longest diameter, NE not evaluable, PFS progression-free survival

In assessed subgroups, including bulky disease, chromosomal deletions, ZAP70 elevation, and immunoglobulin heavy-chain variable region (*IGHV*) mutation status, PFS outcomes favored ibrutinib+BR over placebo+BR (Fig. 2a, Supplementary Figure S3). PFS at 36 months was significantly longer in ibrutinib-treated patients, whether they had one or multiple lines of therapy (Fig. 2b). For patients who had one prior therapy, 36-month PFS was 70.2% in the ibrutinib+BR arm (95% CI: 61.3–77.5) and 15.5% in the placebo+BR arm (95% CI: 8.3–24.7; *P* < 0.0001); for patients who had ≥2 prior therapies, 36-month PFS was 65.9% for ibrutinib+BR (95% CI: 56.8–73.5) and 11.2% with placebo+BR (95% CI: 6.5–17.4; *P* < 0.0001).

Median PFS2 was not reached in either arm but was significantly longer for patients assigned to ibrutinib+BR versus placebo+BR (HR [95% CI], 0.627 [0.445–0.881]; *P* = 0.0067) (Supplementary Figure S2). Among 27 patients who discontinued ibrutinib+BR due to disease progression, 10 patients died (7 patients died due to PD, 2 due to AEs [pneumonia and cardiac arrest] and 1 of unknown causes following administration of subsequent CLL therapy). Eight patients received subsequent systemic CLL therapies, four in combination with rituximab.

**Fig. 3:**
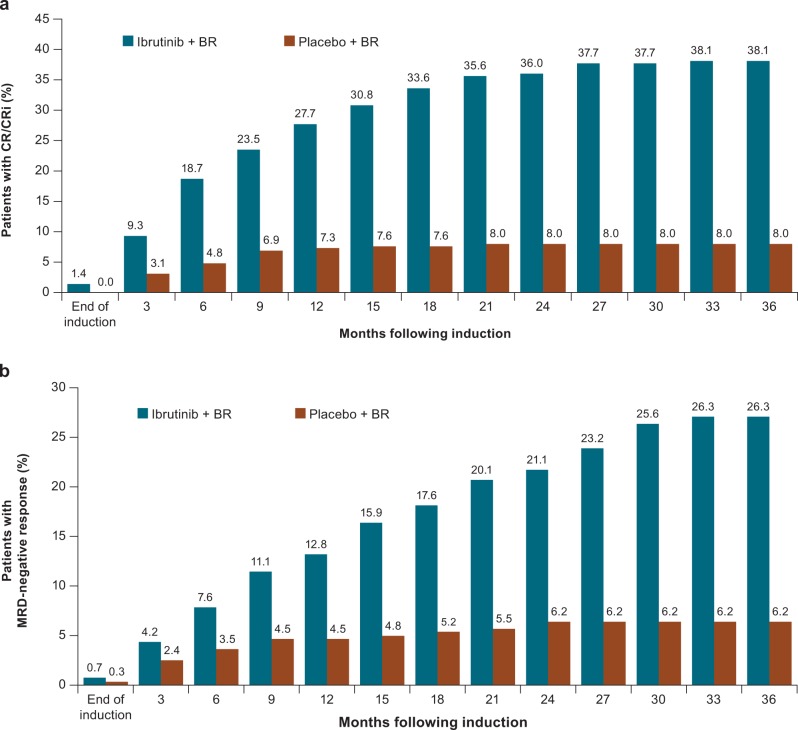
Cumulative response rates over time (investigator-assessed) for **a** complete response and **b** MRD status. Note: The term “induction therapy” refers to BR. The induction phase is defined as the first six cycles of the study, when BR is given along with study drug (ibrutinib or placebo) as combination therapy. The end of the induction phase is the last dose of B or R+30 days. BR bendamustine and rituximab, CR complete response, CRi CR with incomplete bone marrow recovery, MRD minimal residual disease. Note: Percentages are based on the number of patients in the intent-to-treat analysis set in each treatment arm

**Fig. 4:**
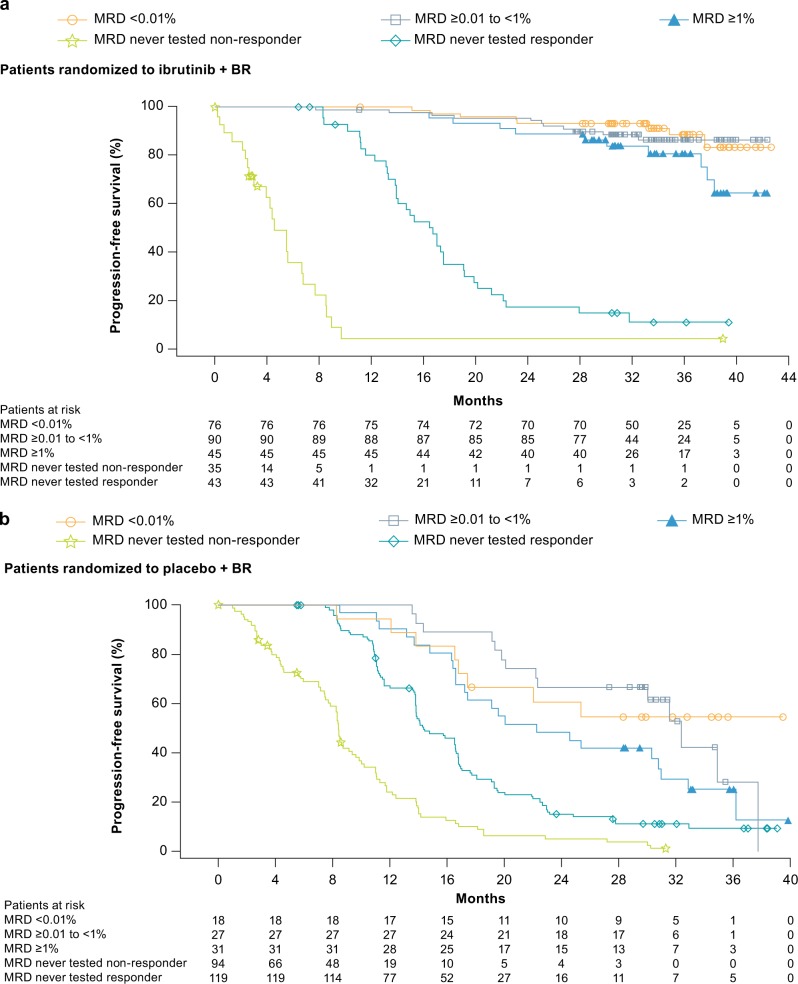
Investigator-assessed PFS by MRD level for **a** ibrutinib+BR and **b** placebo+BR arms. BR bendamustine and rituximab, MRD minimal residual disease, PFS progression-free survival

The investigator-assessed ORR was 87.2% for ibrutinib+BR and 66.4% for placebo+BR (*P* < 0.0001). CR/CRi rates were 38.1% versus 8.0% (Fig. 3a), which showed continued improvement over time versus the investigator-assessed CR/CRi rates of 21.4% and 5.9%, respectively, in the initial analysis [14]. Overall, 211 patients in the ibrutinib+BR arm and 76 patients in the placebo+BR arm were evaluated for MRD; MRD-negative response rates in peripheral blood or bone marrow combined for the intent-to-treat population were 26.3% (76/289) for ibrutinib+BR and 6.2% (18/289) for placebo+BR (*P* < 0.0001) (Fig. 3b). The majority of patients (67.1%) in the ibrutinib+BR arm who achieved MRD negativity had a CR/CRi as their best response; 32.9% patients had a PR as their best response. Of these MRD-negative patients in the ibrutinib+BR arm with PR as their best response, the CR criteria not met are listed in Supplementary Table S3. In the placebo+BR arm, 8/18 MRD-negative patients (44.4%) had PR as their best response. Patients who did not achieve CR/PR or who progressed prior to MRD testing being implemented for all responding patients had a shorter PFS (Fig. 4a, b). Among MRD-evaluated patients, ibrutinib+BR showed a more sustained PFS over placebo+BR at each level of MRD (MRD-negative status <0.01%, HR [95% CI], 0.121 [0.036–0.408], *P* < 0.0001; MRD ≥ 0.01–<1%, HR [95% CI], 0.153 [0.063–0.374], *P* < 0.0001; or MRD ≥ 1–<10%, HR [95% CI], 0.110 [0.035–0.348], *P* < 0.0001) (Fig. 4a, b). In patients receiving ibrutinib+BR, the 36-month PFS rate for MRD-negative patients was 88.6% (95% CI: 76.8–94.6); for those with residual disease (MRD ≥ 0.01%), it was 60.1% (95% CI: 52.6–66.8). In the placebo+BR arm, the 36-month PFS rate in MRD-negative patients was 54.5% (95% CI: 29.2–74.2) and 11.2% (95% CI: 7.1–16.3) for patients with residual disease. A multivariate analysis revealed no difference in OS according to MRD status in responding patients.

### Safety

Following the interim analysis, patients who were randomized to placebo+BR stopped treatment and either crossed over to receive next-line treatment with ibrutinib or remained in follow-up until progression. Per protocol, safety data were collected for 30 days after the last dose of study medication (placebo or BR). Therefore, only safety data for patients randomized to ibrutinib+BR are presented (Table 2); comparison between the two treatment arms up to the interim analysis has previously been published [14]. Treatment-emergent AEs (TEAEs) observed in >10% of patients, and their prevalence over time, are listed in Table 3. The prevalence of TEAEs decreased over time after year 1, except for muscle spasms and hypertension, which remained stable (Table 3).The proportion of patients with all-grade AEs in the ibrutinib+BR arm was 98.3%, with 78.7% of patients reporting grade 3 or 4 events. Grade ≥3 AEs reported in ≥2% of patients are presented in Supplementary Table S4; the most common grade ≥3 AEs were neutropenia (53.7%), thrombocytopenia (15.0%), pneumonia (14.3%), and febrile neutropenia (12.5%), consistent with the initial analysis [14]. Serious TEAEs (i.e., life-threatening, requiring hospitalization, or resulting in persistent/significant incapacity) occurred in 176 (61.3%) patients in the ibrutinib+BR arm; the most common were pneumonia (13.6%) and febrile neutropenia (10.1%). Serious atrial fibrillation (AF) or flutter was reported for 4.9% of patients (compared with 2.8% reporting AF in the initial analysis) [14]. There were 28 (9.8%) TEAEs leading to death in the ibrutinib+BR arm (compared with 19 [6.6%] reported in the initial analysis) [14], of which the most frequent were infections; a complete list of causes are included in Supplementary Table S5.

**Table 2:** Summary of TEAEs in ibrutinib-treated patients

*n* (%)	Ibrutinib+BR (*N* = 287)
TEAEs	282 (98.3)
Grade ≥3	254 (88.5)
Drug related	246 (85.7)
Serious TEAEs	176 (61.3)
Grade ≥3	157 (54.7)
Drug related	102 (35.5)
TEAEs leading to treatment discontinuation	46 (16.0)
TEAEs with outcome of death	28 (9.8)

*BR* bendamustine and rituximab, *TEAE* treatment-emergent adverse event

**Table 3:** Prevalence of most common (≥10% of patients) TEAEs (any grade) for ibrutinib+BR-randomized patients

*n* (%)	0–1 year (*n* = 287)	1–2 years (*n* = 219)	2–3 years (*n* = 188)	3–4 years (*n* = 79)	Overall (*N* = 287)
Patients with any TEAE	278 (96.9)	185 (84.5)	156 (83.0)	62 (78.5)	282 (98.3)
TEAEs reported in ≥10% of patients					
Neutropenia	164 (57.1)	43 (19.6)	9 (4.8)	0	167 (58.2)
Diarrhea	98 (34.1)	39 (17.8)	21 (11.2)	7 (8.9)	110 (38.3)
Nausea	105 (36.6)	9 (4.1)	7 (3.7)	1 (1.3)	106 (36.9)
Thrombocytopenia	86 (30.0)	10 (4.6)	7 (3.7)	3 (3.8)	89 (31.0)
Anemia	64 (22.3)	5 (2.3)	5 (2.7)	1 (1.3)	68 (23.7)
Pyrexia	69 (24.0)	11 (5.0)	6 (3.2)	0	78 (27.2)
Cough	48 (16.7)	25 (11.4)	21 (11.2)	4 (5.1)	65 (22.6)
Fatigue	58 (20.2)	18 (8.2)	16 (8.5)	8 (10.1)	67 (23.3)
Pneumonia	38 (13.2)	20 (9.1)	16 (8.5)	1 (1.3)	61 (21.3)
Upper respiratory tract infection	38 (13.2)	24 (11.0)	10 (5.3)	2 (2.5)	61 (21.3)
Bronchitis	33 (11.5)	15 (6.8)	11 (5.9)	2 (2.5)	50 (17.4)
Sinusitis	22 (7.7)	14 (6.4)	11 (5.9)	0	33 (11.5)
Nasopharyngitis	21 (7.3)	13 (5.9)	4 (2.1)	1 (1.3)	30 (10.5)
Constipation	53 (18.5)	13 (5.9)	14 (7.4)	5 (6.3)	57 (19.9)
Rash	45 (15.7)	23 (10.5)	9 (4.8)	4 (5.1)	56 (19.5)
Infusion-related reaction	48 (16.7)	0	0	0	48 (16.7)
Headache	41 (14.3)	11 (5.0)	8 (4.3)	6 (7.6)	45 (15.7)
Vomiting	40 (13.9)	3 (1.4)	2 (1.1)	0	42 (14.6)
Edema peripheral	32 (11.1)	16 (7.3)	13 (6.9)	7 (8.9)	42 (14.6)
Muscle spasms	34 (11.8)	17 (7.8)	20 (10.6)	7 (8.9)	40 (13.9)
Decreased appetite	35 (12.2)	9 (4.1)	2 (1.1)	0	38 (13.2)
Abdominal pain	30 (10.5)	8 (3.7)	7 (3.7)	2 (2.5)	37 (12.9)
Arthralgia	28 (9.8)	19 (8.7)	11 (5.9)	3 (3.8)	37 (12.9)
Febrile neutropenia	32 (11.1)	2 (0.9)	2 (1.1)	0	36 (12.5)
Back pain	29 (10.1)	8 (3.7)	10 (5.3)	3 (3.8)	36 (12.5)
Hypertension	23 (8.0)	20 (9.1)	20 (10.6)	7 (8.9)	35 (12.2)
Hyperuricemia	27 (9.4)	7 (3.2)	10 (5.3)	3 (3.8)	35 (12.2)
Chills	31 (10.8)	3 (1.4)	2 (1.1)	0	33 (11.5)
Pruritus	29 (10.1)	11 (5.0)	7 (3.7)	2 (2.5)	32 (11.1)

*BR* bendamustine and rituximab, *TEAE* treatment-emergent adverse event

Overall, the incidence of AEs of interest, including cytopenias, bleeding, and infections, reduced during the course of the follow-up period (Table 4). Most AEs occurred within the first 12 months, with a sharp decrease in onset of new events after 12 months. Bleeding events (all grades) were reported in 34.5% of patients in the ibrutinib+BR arm (Table 4) versus 31% of patients in the initial report [14]; most were grade 1/2 events. No new major hemorrhage events or deaths due to bleeding or major hemorrhage events were reported during extended follow-up.

**Table 4:** Incidence of TEAEs of interest by time to new onset for ibrutinib+BR-treated patients

TEAE, *n* (%)	0–1 year (*n* = 287)	1–2 years (*n* = 216)	2–3 years (*n* = 188)	> 3 years (*n* = 83)
Infection	190 (66.2)	22 (10.2)	4 (2.1)	1 (1.2)
Neutropenia	164 (57.1)	3 (1.4)	0	0
Nausea	105 (36.6)	1 (0.5)	0	0
Diarrhea	98 (34.1)	9 (4.2)	1 (0.5)	2 (2.4)
Thrombocytopenia	86 (30.0)	2 (0.9)	1 (0.5)	0
Bleeding	84 (29.3)	10 (4.6)	4 (2.1)	1 (1.2)
Pyrexia	69 (24.0)	5 (2.3)	4 (2.1)	0
Anemia	64 (22.3)	2 (0.9)	2 (1.1)	0
Fatigue	58 (20.2)	5 (2.3)	3 (1.6)	1 (1.2)
Cough	48 (16.7)	12 (5.6)	4 (2.1)	1 (1.2)
Pneumonia	38 (13.2)	15 (6.9)	7 (3.7)	1 (1.2)
Upper respiratory tract infection	38 (13.2)	17 (7.9)	4 (2.1)	2 (2.4)
Hypertension	27 (9.4)	8 (3.7)	4 (2.1)	0
Atrial fibrillation/flutter	19 (6.6)	4 (1.9)	6 (3.2)	0

*TEAE* treatment-emergent adverse event

Ibrutinib therapy is generally well tolerated but has been associated with AF. A detailed review of AF following ibrutinib treatment in HELIOS and other randomized clinical trials investigating ibrutinib has been recently published [20]. During extended follow-up, 8 additional patients in the ibrutinib+BR arm developed AF/flutter, for a total of 29 patients (10.1%). The majority of AF events (17/29) during the entire study duration in the ibrutinib+BR arm were grade 1/2. While dose interruption was normal in these cases, none required dose reductions and none were fatal; 4 (1.4%) led to treatment discontinuation.

Patients randomized to placebo+BR who crossed over to the ibrutinib+BR arm did not demonstrate any difference in type or incidence of AEs compared with patients originally randomized to ibrutinib+BR (Supplementary Table S6).

## Discussion

The HELIOS study was conducted in patients with relapsed/refractory CLL/SLL and is the first trial to show a survival benefit with ibrutinib-based therapy versus a standard chemoimmunotherapy regimen, even in the context of a crossover design. These results support the continued use of ibrutinib, with maintenance of superior PFS and OS versus the placebo+BR arm and an increase in ORR and CR rates over time. It is notable that longer-term follow-up revealed a significant improvement in survival for ibrutinib+BR-treated patients compared with placebo+BR, despite the possibility of crossover after progression. Additionally, deeper responses were reported with continuous ibrutinib therapy, with rates of investigator-assessed CR/CRi and MRD-negative response rising to 38% and 26%, respectively (compared with IRC-assessed rates of 21% and 13% at the primary analysis) [14]. This finding is consistent with the phase 1b study 1108 of ibrutinib+BR, in which CR rates increased from 17% to 40% with 15.7–35.4 months of follow-up, respectively [13].

Among those tested for MRD, patients in the ibrutinib+BR arm demonstrated prolonged PFS compared with those in the placebo+BR arm at the same MRD level. Caution is warranted in interpreting the MRD analyses due to the relatively small numbers of MRD-tested patients in the placebo+BR arm and the potential that longer-term follow-up will be required to fully understand the prognostic significance of specific MRD levels in ibrutinib+BR-treated patients.

The evolution of ORR and of CR rates following ibrutinib monotherapy in study 1102 for treatment-naive (ORR, 71–84%, CR 13–23%, at 22 months to 3 years of follow-up) or previously treated (ORR, 71–90%, CR 2–7% from 26 months to 3 years of follow-up) CLL/SLL patients demonstrates that ibrutinib is associated with durable and deep responses as treatment continues [21]. The results from the HELIOS study have further shown that, in patients with relapsed/refractory disease, an induction-type period of ibrutinib+BR therapy followed by continued ibrutinib treatment produces better responses than BR therapy alone and improves outcomes as the duration of therapy increases [14]. The extended follow-up further confirmed that the positive effects on PFS of continuing ibrutinib following ibrutinib+BR are maintained irrespective of the number of prior lines of therapy or the presence of poor prognostic factors.

It remains unclear whether ibrutinib+BR provides benefits beyond those observed with ibrutinib monotherapy. In the RESONATE trial, which investigated ibrutinib monotherapy in patients with CLL, the 3-year PFS and OS rates for ibrutinib were 59% and 74%, respectively. In our study, 3-year PFS and OS rates for the ibrutinib+BR arm were 68% and 82%, respectively. However, cross-trial comparisons are notoriously difficult to interpret and firm conclusions generally impossible to reach due to potential differences in study designs and treatment populations (e.g., HELIOS did not enroll patients with deletion 17p); an indirect treatment comparison of the HELIOS and RESONATE trials (ibrutinib+BR versus ibrutinib arms respectively) following adjustment for known confounders has recently been published [22]. At a median follow-up of 17 and 19 months, respectively, there was no difference in median PFS or OS, suggesting that addition of BR to ibrutinib does not improve outcomes compared with single-agent ibrutinib. An ongoing study directly comparing BR, ibrutinib+rituximab, and ibrutinib alone in treatment-naive CLL patients (clinicaltrials.gov NCT01886872) will provide more insights into the relative efficacy of chemoimmunotherapy versus ibrutinib alone or with rituximab.

Importantly, the extended follow-up data supported the manageable safety profile of ibrutinib, allowing for continued dosing following the initial induction with BR. The pattern and incidence of AEs and TEAEs was similar to the initial analysis when treatment extended beyond 17 months [14] and was comparable with the safety profile reported in other clinical trials of ibrutinib in CLL patients [5, 14, 21, 23]. Eight additional patients in the ibrutinib+BR arm reported AF/flutter during follow-up, consistent with reviews and meta-analyses documenting an increased risk of developing AF in ibrutinib-treated patients versus comparator treatments [20, 24] and an elevated risk over time [20]. It has previously been reported that 5–9% of CLL/SLL patients receiving ibrutinib are affected [25]. The incidence of bleeding events increased slightly with continued follow-up in the ibrutinib+BR arm; however, there were no new major hemorrhagic events or bleeding-related deaths. These long-term follow-up data support improved survival outcomes with ibrutinib+BR compared with BR alone in relapsed CLL/SLL. In addition, continued ibrutinib monotherapy following the end of chemoimmunotherapy results in continuing improvement in the depth of remission.

## Electronic supplementary material

Supplementary Materials
