# Supplementary material for: Updated results from the phase 3 HELIOS study of ibrutinib, bendamustine, and rituximab in relapsed chronic lymphocytic leukemia/small lymphocytic lymphoma
Source: Leukemia. 2018 Oct 12;33(4):969–80. doi: 10.1038/s41375-018-0276-9 (PMC6484712; doi:10.1038/s41375-018-0276-9)
Supplement: Supplementary file 1 — Supplementary Materials [file 41375_2018_276_MOESM1_ESM.docx]

**Supplementary Tables and Figures**

**Supplementary Table S1.** Ibrutinib exposure by yearly intervals.

|  | **0–1 year**  **(*n*=287)** | **1–2 years**  **(*n*=216)** | **2–3 years**  **(*n*=188)** | **3–4 years**  **(*n*=83)** | **Overall**  **(*N*=287)** |
| --- | --- | --- | --- | --- | --- |
| **Patients in each year of treatment, *n* (%)** | 0 | 1 (0.6) | 91 (71.1) | 79 (95.2) | 171 (29.6) |
| **Median (range) treatment duration, months** | 31.7  (0–43) | 33.7  (12–43) | 35.2  (24–43) | 39.3  (36–43) | 34.7  (0–43) |
| **Median (range) daily dose, mg/day** | 408.0  (174–420) | 408.7  (174–420) | 410.5  (201–420) | 408.6  (201–420) | 408.7  (174–420) |

**Supplementary Table S2.** Baseline characteristics.

|  | Ibrutinib+BR  (*N*=289) | Placebo+BR  (*N*=289) |
| --- | --- | --- |
| Median age, years (range) | 64 (31–86) | 63 (36–83) |
| Male, *n* (%) | 193 (66.8) | 189 (65.4) |
| Diagnosis, *n* (%) |  |  |
| CLL | 257 (88.9) | 257 (88.9) |
| SLL | 32 (11.1) | 32 (11.1) |
| ECOG performance status, *n* (%) |  |  |
| 0 | 125 (43.3) | 126 (44.6) |
| 1 | 164 (56.7) | 163 (56.4) |
| Rai stage, *n*^a^ | 256 | 258 |
| 0–II*, n (%)* | 157 (61.3) | 139 (53.9) |
| III–IV*, n (%)* | 99 (38.6) | 119 (46.1) |
| Binet stage, *n*^a^ | 256 | 258 |
| A*, n (%)* | 26 (10.2) | 23 (8.9) |
| B*, n (%)* | 132 (51.6) | 119 (46.1) |
| C*, n (%)* | 98 (38.3) | 116 (45.0) |
| Bulky disease ≥5 cm, *n* (%) | 168 (58.1) | 156 (54.0) |
| Del11q*, n (%)* | 87 (30.1) | 65 (22.5) |
| *IGHV* status, *n*^a^ | 259 | 260 |
| Mutated, *n* (%) | 49 (18.9) | 52 (20.0) |
| Unmutated, *n* (%) | 210 (81.1) | 208 (80.0) |
| ZAP70 expression, *n*^a,b^ | 271 | 276 |
| Elevated, *n* (%) | 204 (75.3) | 190 (68.8) |
| Not elevated, *n* (%) | 67 (24.7) | 86 (31.2) |
| Purine analogue refractory, *n* (%) | 75 (26.0) | 74 (25.6) |
| Previous lines of therapies | 289 | 288 |
| Mean (range) | 2 (1–11) | 2 (1–9) |
| 1, *n* (%) | 140 (48.4) | 138 (47.9) |
| 2, *n* (%) | 72 (24.9) | 78 (27.1) |
| ≥3, *n* (%) | 77 (26.6) | 72 (25.0) |
| Previous therapy, *n* (%) |  |  |
| Purine analogue | 206 (71.3) | 209 (72) |
| Alkylating agent | 275 (95.2) | 275 (95.2) |
| Anti-CD20 mAb | 203 (70.2) | 200 (69.2) |
| Common regimens used*, n (%)* |  |  |
| FCR | 120 (41.5) | 109 (37.7) |
| Other fludarabine-based combinations | 92 (31.8) | 102 (35.3) |
| BR | 10 (3.5) | 9 (3.1) |
| Chlorambucil+anti-CD20 mAb | 16 (5.5) | 15 (5.2) |
| Time from progression or relapse since last line of treatment to randomization, months (range) | 2.9 (0–48) | 2.6 (0–73) |
| Time from last treatment to randomization, months (range) | 24.0 (0.7–154.8) | 20.9 (0.2–160.8) |

BR, bendamustine and rituximab; CLL, chronic lymphocytic leukemia; ECOG, Eastern Cooperative Oncology Group; FCR, fludarabine, cyclophosphamide, and rituximab; *IGHV*, immunoglobulin heavy-chain variable region; mAb, monoclonal antibody; SLL, small lymphocytic lymphoma.

^a^Staging criteria for patients with chronic lymphocytic leukemia only, using diagnosis at study entry; not all samples were evaluable for biomarker data.

^b^ZAP70 levels above cutoff results (69%) are reported as “elevated.”

**Supplementary Table S3.** Reasons CR not met in MRD-negative patients with PR as best response.

| **Unmet criteria, *n* (%)** | **Ibrutinib+BR**  **(*N*=289)** |
| --- | --- |
| MRD-negative patients with PR as best response | 25 (8.7) |
| Unresolved lymphadenopathy and/or organomegaly (spleen or liver) | 15 (5.2) |
| Met clinical criteria; no confirmatory bone marrow data | 4 (1.4) |
| CBC did not meet CR criteria | 3 (1.0) |
| Progression shortly after MRD-negative response | 2 (0.7) |
| Persistent disease-related symptoms | 1 (0.3) |

BR, bendamustine and rituximab; CBC, complete blood count; CR, complete response; MRD, minimal residual disease; PR, partial response.

**Supplementary Table S4**. Summary of most common (≥2%) grade 3 or worse AEs in the ibrutinib+BR arm.

| ***n* (%)** | **0–1 year**  **(*n*=287)** | **1–2 years**  **(*n*=219)** | **2–3 years**  **(*n*=188)** | **3–4 years**  **(*n*=79)** | **Overall**  **(*N*=287)** |
| --- | --- | --- | --- | --- | --- |
| Patients with any grade ≥3 TEAE | 238 (82.9) | 96 (43.8) | 58 (30.9) | 15 (19.0) | 254 (88.5) |
| Neutropenia | 151 (52.6) | 27 (12.3) | 7 (3.7) | 0 | 154 (53.7) |
| Thrombocytopenia | 42 (14.6) | 5 (2.3) | 0 | 0 | 43 (15.0) |
| Pneumonia | 26 (9.1) | 7 (3.2) | 10 (5.3) | 1 (1.3) | 41 (14.3) |
| Febrile neutropenia | 32 (11.1) | 2 (0.9) | 2 (1.1) | 0 | 36 (12.5) |
| Neutrophil count decreased | 15 (5.2) | 2 (0.9) | 1 (0.5) | 0 | 17 (5.9) |
| Hypertension | 7 (2.4) | 7 (3.2) | 7 (3.7) | 3 (3.8) | 13 (4.5) |
| Atrial fibrillation | 8 (2.8) | 2 (0.9) | 3 (1.6) | 0 | 12 (4.2) |
| Anemia | 10 (3.5) | 0 | 1 (0.5) | 0 | 11 (3.8) |
| Bronchitis | 7 (2.4) | 1 (0.5) | 3 (1.6) | 1 (1.3) | 10 (3.5) |
| Fatigue | 7 (2.4) | 2 (0.9) | 1 (0.5) | 0 | 10 (3.5) |
| Leukopenia | 10 (3.5) | 1 (0.5) | 0 | 0 | 10 (3.5) |
| Pyrexia | 10 (3.5) | 1 (0.5) | 0 | 0 | 10 (3.5) |
| Tumor lysis syndrome | 10 (3.5) | 0 | 0 | 0 | 10 (3.5) |
| White blood cell count decreased | 9 (3.1) | 1 (0.5) | 0 | 0 | 9 (3.1) |
| Platelet count decreased | 7 (2.4) | 0 | 1 (0.5) | 0 | 8 (2.8) |
| Sepsis | 6 (2.1) | 2 (0.9) | 0 | 0 | 8 (2.8) |
| Hyperuricemia | 7 (2.4) | 0 | 0 | 0 | 7 (2.4) |
| Lymphopenia | 7 (2.4) | 4 (1.8) | 1 (0.5) | 0 | 7 (2.4) |
| Urinary tract infection | 4 (1.4) | 3 (1.4) | 1 (0.5) | 1 (1.3) | 7 (2.4) |
| Cellulitis | 5 (1.7) | 0 | 1 (0.5) | 0 | 6 (2.1) |
| Diarrhea | 6 (2.1) | 0 | 0 | 0 | 6 (2.1) |
| Upper respiratory tract infection | 6 (2.1) | 0 | 0 | 0 | 6 (2.1) |

AE, adverse event; BR, bendamustine and rituximab; TEAE, treatment-emergent adverse event.

**Supplementary Table S5.** Causes of death in patients randomized to ibrutinib+BR by system organ class.

| **Cause, *n* (%)** | **Ibrutinib+BR**  **(*N*=287)** |
| --- | --- |
| Total deaths | 28 (9.8) |
| Infections and infestations | 9 (3.1) |
| Septic shock | 3 (1.0) |
| Progressive multifocal leukoencephalopathy | 2 (0.7) |
| Sepsis | 1 (0.3) |
| Cytomegalovirus infection | 1 (0.3) |
| Fungal infection | 1 (0.3) |
| *Pneumocystis jirovecii* pneumonia | 1 (0.3) |
| Gastrointestinal disorders | 1 (0.3) |
| Large intestine perforation | 1 (0.3) |
| General disorders and administration site conditions | 8 (2.8) |
| Death | 2 (0.3) |
| Disease progression | 1 (0.3) |
| General physical health deterioration | 1 (0.3) |
| Multi-organ failure | 1 (0.3) |
| Sudden cardiac death | 1 (0.3) |
| Sudden death | 1 (0.3) |
| Systemic inflammatory response syndrome | 1 (0.3) |
| Respiratory, thoracic and mediastinal disorders | 2 (0.7) |
| Lung infiltration | 1 (0.3) |
| Respiratory failure | 1 (0.3) |
| Injury, poisoning and procedural complications | 1 (0.3) |
| Post-procedural hemorrhage | 1 (0.3) |
| Vascular disorders | 1 (0.3) |
| Aortic aneurysm rupture | 1 (0.3) |
| Cardiac disorders | 4 (1.4) |
| Cardiac arrest | 1 (0.3) |
| Cardiac failure | 1 (0.3) |
| Cardiopulmonary failure | 1 (0.3) |
| Ventricular flutter | 1 (0.3) |
| Neoplasms benign, malignant and unspecified (including cysts and polyps) | 3 (1.0) |
| Myelodysplastic syndrome | 2 (0.7) |
| Chronic myelomonocytic leukemia | 1 (0.3) |

BR, bendamustine and rituximab.**Supplementary Table S6.** Summary of TEAEs (≥10%) during crossover to ibrutinib therapy.

| ***n* (%)** | **Placebo + BR patients receiving  crossover ibrutinib therapy**  **(*n*=160)** |
| --- | --- |
| TEAEs | 134 (83.8) |
| Grade ≥3 | 81 (50.6) |
| Drug-related | 97 (60.6) |
| Serious TEAEs | 62 (38.8) |
| Grade ≥3 | 51 (31.9) |
| Drug-related | 28 (17.5) |
| TEAEs leading to treatment discontinuation | 10 (6.3) |
| TEAEs with outcome of death | 15 (9.4) |

BR, bendamustine and rituximab; TEAE, treatment-emergent adverse event.

**Supplementary Figure S1.** OS corrected for patient crossover by (**a**) Inverse probability of censoring weighting (IPCW) method and (**b**) Rank Preserving Structural Failure Time (RPSFT) method.


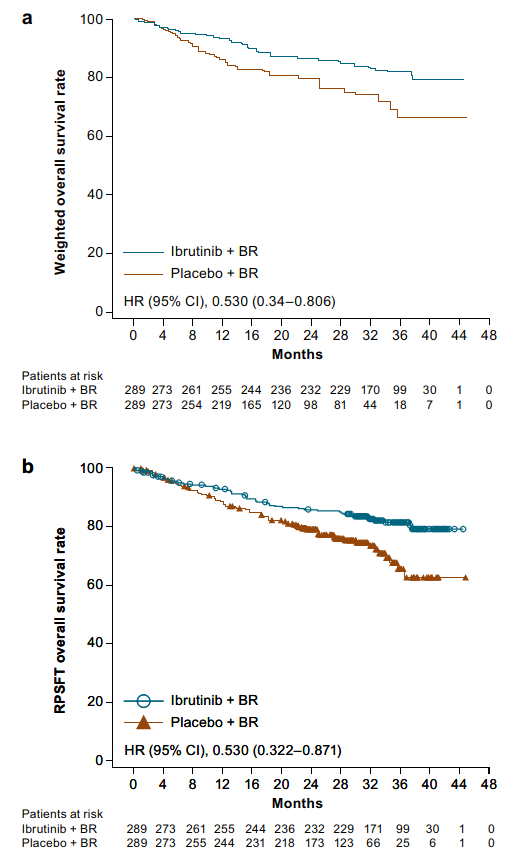


BR, bendamustine and rituximab.

**Supplementary Figure S2.** Investigator-assessed PFS2.


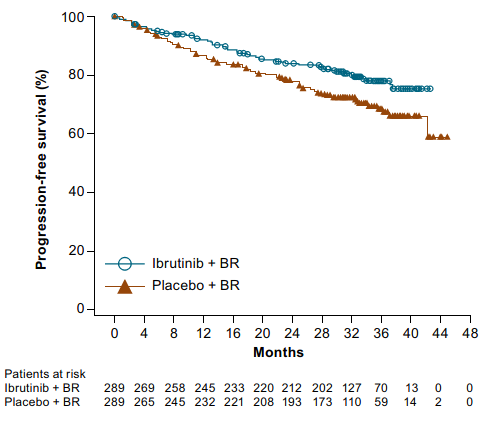


BR, bendamustine and rituximab; PFS2, progression-free survival following next line therapy.

**Supplementary Figure S3.**PFS by (**a**) bulky disease status, (**b**) chromosome 11q deletion status, (**c**) *IGHV* status, (**d**) ZAP70 status, (**e**) complex karyotype, (**f**) trisomy 12 and (**g**) chromosome 13q deletion status.


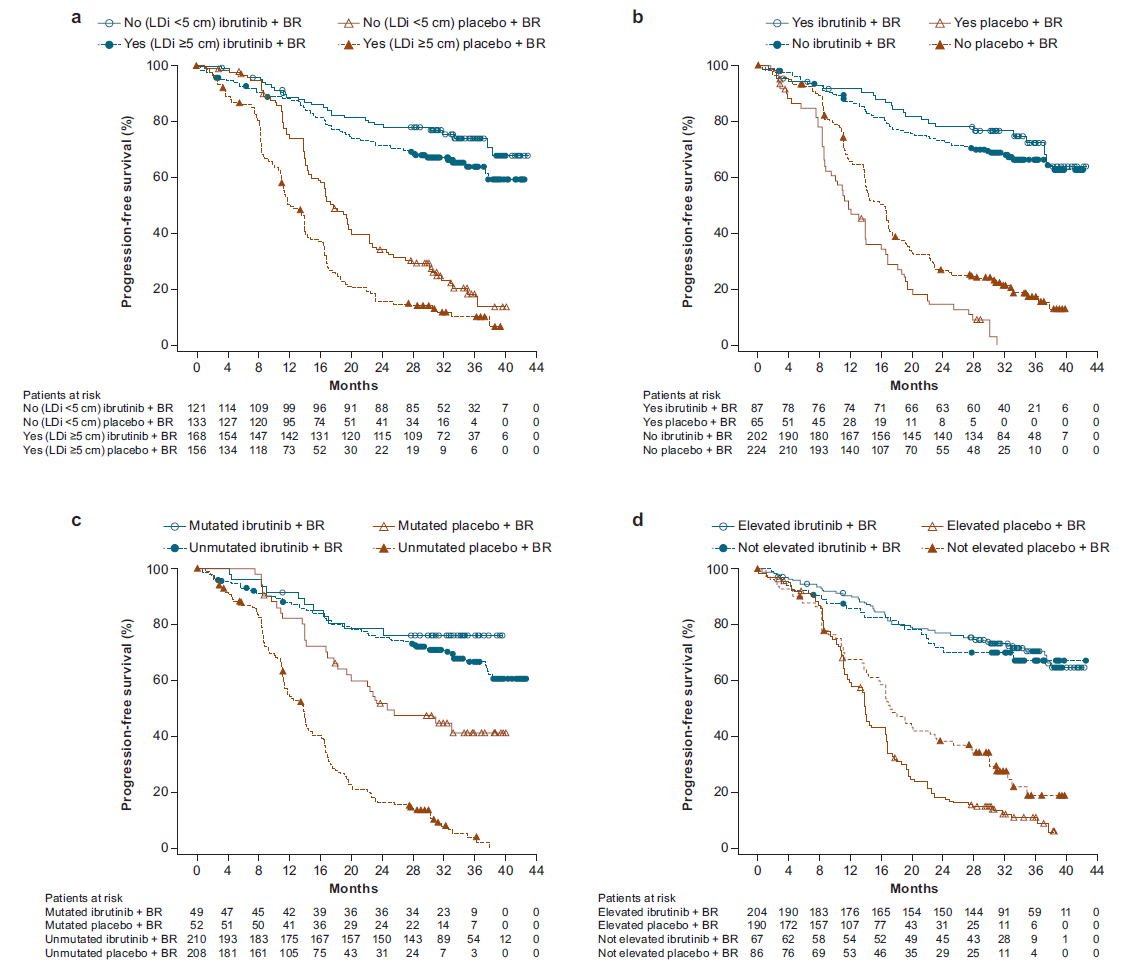


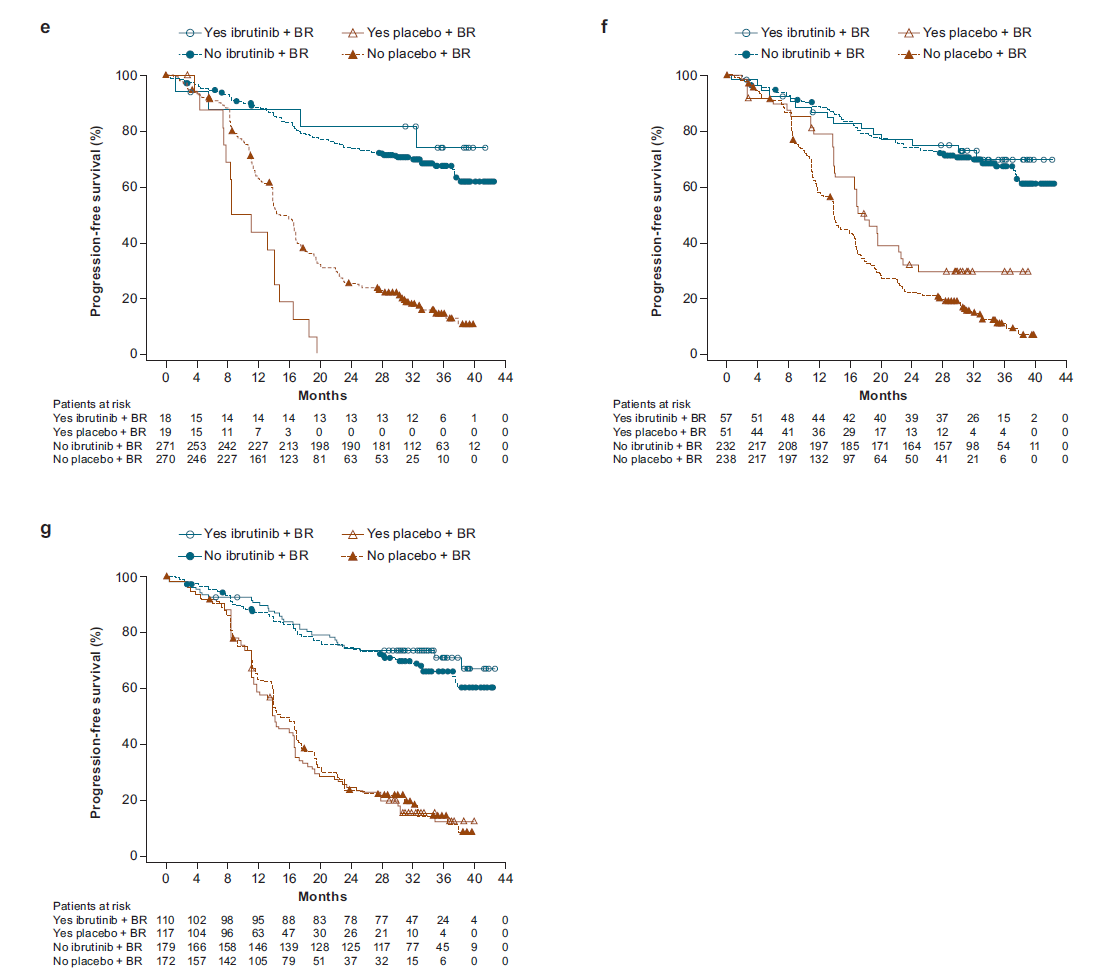


BR, bendamustine and rituximab; *IGHV*, immunoglobulin heavy-chain variable region; PFS, progression-free survival; ZAP70, zeta-chain-associated protein kinase 70.
